# Economics and economists during the COVID-19 pandemic: a personal view

**DOI:** 10.1186/s41937-022-00097-1

**Published:** 2022-11-08

**Authors:** Monika Bütler

**Affiliations:** SEW-HSG, University of St. Gallen, St. Gallen, Switzerland

**Keywords:** COVID-19 pandemic, Economic policy advice, Policy evaluation

## Abstract

As was true for many others, my professional life was turned upside down in the early days of the pandemic. The crisis touched almost every field in economics: international supply chains broke down, economic activity was heavily constrained either by non-pharmaceutical measures to fight the pandemic or by voluntary action, and the labour market experienced unprecedented levels of short-time work and huge (temporary) lay-offs. Governments struggled to provide cash and find ways to compensate affected people and businesses. Financial markets tumbled and monetary policy faced new challenges on top of an already tense situation.

## Introduction

This is not a research paper, nor is it a literature survey on economic aspects of the pandemic. It is my own unbalanced assessment of economics as an academic discipline and the work of economists during the pandemic’s first two years with a clear focus on Switzerland. Why personal? A fascinating research strand, nicely summarized by one of its founders, Ulrike Malmendier (2021), shows that personal experiences of economic events and outcomes, from global crises such as a pandemic to individual experiences such as a job loss, can shape individual expectations and attitudes. These changes in perception, and perhaps even preferences, can impact choices for years to come. It would thus be naive and logically inconsistent to believe that academic economists were untouched by their environment or by their personal experience.

It is thus only fair to disclose my own predisposition: I have always been interested in policy, be it social or economic. When I traded math/physics for economics, the proximity of social aspects was key. Individuals and countries are not machines that can be understood without a context, let alone be programmed. The same is true for medical science, my second lay passion. I wrote my diploma thesis on the progression of AIDS in the late 80 s, with the first available individual-level data on the disease. Last, but not least, I have always kept one foot outside academia. Considering issues from another angle helps to come up with more nuanced views. But even after leaving university, I am still a scientist at heart, an empiricist to be more precise.

In fact, academic research has provided an important backbone for those of us working on public policy during the crisis. Literally, thousands of papers by economists on issues around COVID-19 have mushroomed since the onset of the crisis. The two most important research networks in economics alone, CEPR and NBER, published more than 1000 high-quality COVID-19-related research papers in the first 15 months of the pandemic. While the frequency of new contributions is somewhat petering off with the research on the pandemic maturing, there will be many more to come. This journal—with its Special Focus on COVID-19—has done an excellent job in publishing papers that are based on sound economic research but cover topics that are relevant to (Swiss) economic policy (Tille et al., 2022).

In this paper, I look at the role of economics as an academic discipline and academic economists during the pandemic. Starting from the efforts to shed light on the economic aspects of the crisis in research, I will comment on the challenges economists faced when directly involved in policy advice and working in interdisciplinary groups of academics and under time pressure. Two important qualifications: To summarize academic economists’ contribution during the pandemic would break the mould of such an essay. The paper thus has a clear focus, or call it bias, first, on Switzerland and, second, on public work done by economists in the Swiss National COVID-19 Science Task Force (ncs-tf).

The first part of the paper focuses on the “how”. After a short overview of the research efforts related to COVID-19, I describe the role of academic economists in firefighting teams such as the Swiss National COVID-19 Science Task Force, and in informing decision-makers and the public during the pandemic. This was no easy task as interests among the public and within the private sector often conflicted and acceptable trade-offs in terms of measures to fight the pandemic were difficult to be found. In contrast to other academic disciplines involved during the pandemic, however, academic economists had ample experience in working with politics and the administration due to prior exposure to global crises, notably the financial crisis.

The paper’s second part deals with the “what”: It describes, among other things, the importance of data and the sound application of economic principles to answer new questions emerging during the pandemic. Two findings stand out. First, simple economic ideas and tools were extremely useful in understanding the crisis and come up with good ideas for policy. Second, academic economists were very creative in finding new data or new ways to display them and thus contributed in important ways to better policy options.

An essay covering an important and severe, yet ultimately limited, crisis would not be very insightful if it did not lend itself to learnings that survive the pandemic. I will try to comment on potential improvements for (policy) work by economists throughout the paper and summarize these lessons in my conclusions. One important lesson is that academic scholars—be it in economics or other fields—should not be punished for providing public goods such as communicating to the public, or analysis not directly publishable in reputable journals. On the contrary: we need ways to support internationally respected scholars in venturing out of the ivory tower for certain periods in their career.

## Getting on stage: from research to economics for the public

It is perhaps a bit unconventional to start with the “how” instead of the “what”. But to understand the role of economists during the crisis it helps to see in what areas they have been active.

## Research as the backbone for policy advice

In the absence of a major pandemic for over a century, relatively little research on the economics of pandemics existed that could be taken from the shelf. A few papers on the Spanish flu offer insights into the nature of health–wealth trade-offs (see, for example, Correia et al. (2020)). Other work explores a pandemic’s long-run consequences on economic outcomes (such as Almond (2006)). Related to the latter—and potentially very important to understand the (economic) long-run effects of COVID-19—a number of papers look for and find an impact of early life health shocks on labour market outcomes and other variables (Almond et al., (2018) and literature cited therein).

The lack of research did not last long, as Charles Wyplosz, the editor of Covid Economics, remarked:

*Within days of the onset of Covid, hundreds of economists dropped their ongoing work. They relied on well-established theories and techniques and on quickly expanding real-time data to fill a vacuum in the well-established field of epidemiology. (*Wyplosz, 2021*, p.1)*

And further: *“Epidemiologists tracked viruses, economists looked at people behaviour and government responses.” (*Wyplosz, 2021*, p.1)* Which is not quite correct, of course. Economic activity and the spread of viral diseases interact, as Adda (2016), among others, had pointed out well before the crisis. During the pandemic, many economics papers also ventured into epidemiologic modelling. While some economists may have welcomed new questions primarily for the sake of research in a pretty saturated environment, most were truly concerned and spent time and effort on questions with an unclear publication prospect.

Two institutions, NBER and CEPR, stood out in collecting COVID-19-related research in a systematic way, ensuring quick dispersion of new findings while maintaining the quality through different methods. NBER limits contributors through membership, selected through a rigorous and competitive process of researchers mainly from top US departments. It lists approximately 550 papers (until March 2022) sorted by topic area for easy access. Very helpful are two additional categories listed by the NBER repository: papers on the 1918 Spanish Flu and other pandemics, as well as selected pre-2020 papers of related interest.

CEPR followed a different approach. The platform, usually reserved for the network’s affiliates and fellows, opened up for submissions by authors from all over the world, including students and faculty in lesser-known departments. The contributions were vetted by editors for quality and relevance. In contrast to refereeing, vetting does not offer the possibility of revising and resubmitting; the paper is directly accepted or rejected. Authors retain copyright and are free to submit to established outlets later. The papers were collected in volumes and published free of charge online. Through this process, the real-time nature of research on COVID-19 was adequately mirrored and the accepted research appeared online a few days after submission.

From March 2020 to June 2021, the editorial team of CEPR around Charles Wyplosz received close to 1200 submissions, out of which 511 papers were collected in 83 issues. CEPR closed the platform due to the maturity of the field and the usual submission process was re-established after June 2021. Nonetheless, the lessons learnt from this innovative process will certainly shape the publication process in the future. In a recent paper, Charness et al. (2022) present survey evidence that economists indeed want to see changes to the peer review system in that direction.

At this stage, the impact of these papers on economic policy is difficult to evaluate. It remains to be seen, what fraction of created knowledge has found or will eventually find its way to economic policy, and which holes were left open.

### Part of the firefighting team: economists as part of the scientific task force

Economics was not viewed as a key discipline for the fight against the pandemic in February 2020. The first science task force, organized by the two federal institutes of technology (ETHZ and EPFL), did not include economists despite the presence of such scholars at both affiliated universities. When Matthias Egger, the science task force’s first president, set up the interdisciplinary group of experts at the end of March 2020; however, economists were on board with a dedicated own expert group. By then, the interdisciplinary nature of the crisis had become obvious, with strong interdependencies between economic policy and public health measures such as the closure of schools or restaurants and restrictions to businesses and public transport.

Whether real or alleged, scepticism from other scientists quickly disappeared. But it also became clear that there was a fundamental misunderstanding of what economics as an academic discipline really means. By many, economics was equated with a vague concept of what they perceived as “the economy”, largely big firms, often with a negative connotation. Tellingly, the first name of the economists’ expert group was economy, not economics. Sometimes, economics was also perceived as working in the interest of businesses fighting for their own good. It had to be repeated over and over again: Economics is not business.

Like other ncs-tf expert groups, the economics group was criticized as non-representative for the economic expertise related to the crisis. Especially during the second wave, some think tanks and politicians asked for “real-world” (non-academic) economists to be included in the task force. Interestingly, most of these proposals concerned economists in associations that had ample opportunity to voice their expertise or opinion in public or to decision-makers. Moreover, as the members of the ncs-tf contributed their time and energy pro bono, the entry hurdle for other economists, be it in academia or outside, to participate in the public debate was low. As far as I can judge as an insider, the spectrum of views on policy within the economists group mirrored the breadth of the economic research during this period pretty well. And science was the common denominator of the ncs-tf after all.

To understand the context, a few words on the ncs-tf: The independent expert group was active from April 2020 to the end of March 2022. In its most active period, the ncs-tf consisted of approximately 80 experts in ten expert groups, with a management team of four responsible for coordinating and communicating with the public. Experts participated completely voluntarily and were not remunerated for their work in the ncs-tf by the Federal Government or third parties. (For further reference and additional details, see ncs-tf (2022, March 29).)

The ncs-tf followed an official mandate by the Federal Office of Public Health (FOPH) and the Federal Department of Home Affairs (FDHA). Its scientific knowledge should assist the political authorities and decision-makers—federal authorities and cantonal administrations—in reaching decisions. The ncs-tf’s goals consisted of, among others, providing scientific support for the development of an effective surveillance-response strategy, crucial for containing COVID-19 and thus preventing major damage to people’s health or the economy. The ncs-tf also made large efforts to support the collection and analysis of data on the pandemic. It provided assistance in finding effective vaccination and treatment strategies to overcome the crisis. Last, but not least, the ncs-tf aimed at understanding the economic and social context of the crisis to help minimize its damage to the economy and society.

It is important to underline that the principal of the mandate was the health authorities in Switzerland, mainly the FOPH. Despite the crisis covering all aspects of society, other federal departments, notably the State Secretariat for Economic Affairs (SECO) and the Federal Finance Administration were left aside. While economic and social aspects did play a role also in conversations with the FOPH, the economists thus had a somewhat limited scope. Questions of the authorities addressed to the ncs-tf did not include the broad range of topics covered in the real world or research. But of course, economists were free to venture out of the mandate’s range—which they did in a number of public policy papers.

To some degree, the limits of the mandate were reflected in the composition of the economics expert group. As the first chair of the group, I was part of the selection process together with the task force’s first president Matthias Egger. In the very short time frame in which we had to choose the names, we gave preference to economists with some affinity to public health questions and previous experience with policy work. The expert group economics was, like other expert groups, never thought to be a closed group. New colleagues joined later, some left for other tasks. From the beginning, other economists were incorporated for specific topics.

Within the ncs-tf, economic experts were taken seriously and listened to from the start. Economic analysis and data, as well as economic concepts such as incentives and externalities, were met with interest and taken into account in the discussions within the ncs-tf. Economic aspects of the pandemic started to play a larger role in the task force’s policy outlets and communications. From an early phase on, economists were invited as part of the ncs-tf to the federal administration’s emergency task force (Krisenstab) and were frequent presenters at the federal administration’s COVID-19-related press conferences (points de presse). Starting from July 2020 to the end of the formal ncs-tf, one economist was always part of the ncs-tf’s management team of four.

The expert group economics met once or twice per week over zoom, most economists also participated in the plenum’s meetings that took place up to three times a week. The expert group drafted 16 policy briefs as sole or main contributors and participated in many more outlets of the ncs-tf, mainly directed at the FOPH and, ultimately, the public. Table 1 presents a list of these policy briefs including a short summary of the questions addressed.

**Table 1:** ncs-tf policy briefs (co-)written by economists (ncs-tf, n.d.b)

Title	Value judgements? bottom line?	Date published	Main field	Trade-offs discussed
Contact tracing costs	Benefits of contact tracing outweighs its costs	24/04/2020	Macro, public	Cost of contact tracing vs. Easing of lockdown/opening businesses
Public matching payments for commercial rent abatements during the COVID-19 crisis	Proposal: government could match rent abatements to incentivize landlords not to dissolve rental contracts	01/05/2020	Public	
Economic considerations of test-isolate-trace-quarantine (TITQ)	Federal government as a single-payer to incentivize testing. Quarantined people need to be financially and legally protected	10/05/2020	Public	Private and social costs and benefits of testing/isolating
Comparison of Sweden and Switzerland	Comparison of economic and epidemiological indicators between Switzerland and Sweden	13/05/2020	Macro + oTF	Mandatory closures vs. Voluntary measures
How to repay the government debt resulting from the COVID-19 crisis?	Proposal: Dept repayment over a longer time period than 6 years (i.e. 30 years)	20/05/2020	Public	Repaying debt swiftly vs. slowdown of economic activity due to spending cuts
Disruption of the Swiss labour market: 2020 Corona crisis and 2008 Financial crisis compared	Short-term work for sectors that are affected long-term prolonged, but not permanently	16/06/2020	Labour	
Digital proximity tracing—the view from economics	Different nudging recommendations and potential incentives to implement so that people install the app	10/07/2020	Behavioural + oTF	Overestimation of costs and underestimation of benefits from downloading the SwissCovid app
Tackling weak investment with an adjustment to the COVID-19 credit programme	Proposal: COVID-19 credit programme to be prolonged and adjusted, such that the loans can also be used for investment, not just operational costs	03/08/2020	Macro, Public	Supporting investment vs. supporting non-viable projects (aka zombies)/risking federal budget deficits due to loan defaults
Is there a health–wealth trade-off during the COVID-19 crisis?	Some interventions are necessary and economic recovery only possible with virus containment. Testing and quarantining more important than lockdowns, economic costs can be minimized through publicly funded support measures	18/08/2020	Macro	Health vs. wealth
Widespread community spread of SARS-CoV-2 is damaging to health, society and the economy	Community infection would lead to massive damage to health, economy and society	14/09/2020	Entire TF	Health vs. wealth
The rationale for a substantial increase in resources for contact tracing and testing	Proposal: federal funding (money and manpower) of an increase in testing capacity, make testing free for all, promote testing in the population	26/10/2020	Public	Costs of lockdown vs. costs of testing/contact tracing
Estimating the economic costs of avoiding COVID-19 transmission through quarantine and testing of travellers arriving in Switzerland	Travel quarantine is expensive compared to contact tracing	08/11/2020	Macro	Economic activity through increased travel vs. more infections
Support to businesses in the second COVID-19 wave 1	Proposal to reactivate COVID-19 credits	10/11/2020	Public	Fiscal support to capital owners vs. keeping alive non-viable firm
Warum aus gesamtwirtschaftlicher Sicht weitgehende gesundheitspolitische Massnahmen in der aktuellen lage sinnvoll sind	Health measures should be continued (but are only needed for a limited time), as their benefits outweigh the costs in economic terms	19/01/2021	Macro	Health vs. wealth
Die wirtschaftlichen Vorteile einer beschleunigten Impfkampagne	Speeding up vaccination campaign has large benefits (at least CHF 25Mio for advancing start by one day)	15/03/2021	Macro	
Auswirkung des Zertifikats auf das Gastgewerbe	Estimates of economic impact of COVID-19 certificates on restaurants	08/11/2021	Labour, Business	Effect of certificates on restaurants/bars

In the course of the pandemic, it proved to be an advantage that many of the ncs-tf economists had prior experience both in working with decision-makers and communicating to the public. Previous crises helped to establish links between policymakers and academic economists, facilitating a smoother transition from academic work to policy advice. Another factor that facilitated the dialogue with the authorities can be found in a sizeable number of academically trained economists in leading positions outside universities, notably at the SECO and the Swiss National Bank (SNB).

### Educating and informing the public and policymakers

The involvement of academic economists in coping with the crisis went well beyond research efforts and a direct involvement in the ncs-tf, of course. Even before formal institutions were formed, the more publicly visible or better-known economists were recruited for interviews and broadcasts. Many more participated with ad hoc expert groups commissioned by the federal and cantonal administrations. An exhaustive list of engagements of academic economists would go well beyond the scope of this article, but in what follows I give some examples of their work in Switzerland.

The media work of economists during the pandemic was extensive. Both members of the ncs-tf and others participated in the public domain and provided additional insights into their respective domains of expertise. They met a large interest in assessing the economic costs of and compensation policies for restricting measures as well as with international distortions, but also in finding the right balance between restrictions and letting the economy run more freely. Some universities bundled the media work of their academics for easy reference (see for example UBS Center (2022)).

Of course—and fortunately—academic economists did not speak with one voice. A broad range of topics and opinions can be found among the media contributions, many differing from the analysis and consensus positions of the ncs-tf members. Examples of more controversial and contested inputs were proposals to boost society’s immunity through a controlled infection strategy at the start of the pandemic, and the idea to use immunity certificates to facilitate the restart of the economy (Eichenberger et al., 2020).

Apart from traditional media outlets, blogs helped to quickly disseminate early analysis and provided an outlet for evidence-based contributions, some of which were taken up by the media or published later in revised form in established journals. One example is regional estimates of the possibility to resort to home office, as an early indicator of how intensely the shutdown will be felt (Faber et al., 2020a). Another one is the use of a readily available short-time work calculator as a means to estimate the extent to which different regions are affected by short-time work (Faber et al., 2020b).

Open letters, position papers, or appeals to act constituted an alternative way to reach policymakers and the public. While not used by economists very often in the past, they generated a high resonance during the pandemic. At the beginning of the crisis, on 26 March 2020, a position paper signed by all (!) professors of the University of Zürich’s (UZH) Department of Economics described the consensus emerging in the economic discipline at that time and offered advice on what UZH’s economists thought this meant for Switzerland. Two proposals were made: frequent and broad testing as well as freezing the economy for a number of weeks (UZH, 2020). The UZH’s position paper mirrored similar ones in other countries. Retrospectively, the proposal to freeze may sound somewhat mechanistic. Many economists, including myself, potentially underestimated both the flexibility of economic actors to adjust and invent, as well as the long-lasting impact of freezes.

Only a few weeks later, when Switzerland had been in a partial freeze for approximately a month, academic economists and medical doctors from Lausanne and Geneva published a position paper on how to safely exit from a lockdown. The scientists recommended a gradual sectoral exit to avoid exceeding the capacity limits of the hospitals by applying a number of criteria, such as the ability of an industry to also function with "home office", its importance to the national economy, value creation and employment, and social contact intensity of the activities concerned (Bonardi et al., 2020a). Again, similar proposals were made in other countries (Baqaee et al, 2020). A revised version of the Swiss position paper was later published by the Harvard Business Review (Bonardi et al., 2020b).

Probably the largest domestic and international echo—it found its way into several international media outlets including the Financial Times—was generated by an open letter signed by 60 economists in November 2020. By then Switzerland, whose citizens enjoyed a high degree of liberty from restraining measures by international standards, was close to the peak of the second wave, with high levels of mortality and ICU occupancy reaching critical levels. The economists’ open letter was addressed to the government, urging the decision-makers to rethink its coronavirus strategy and impose a nationwide lockdown in view of soaring COVID-19 cases. The relatively short letter also reiterated the widely accepted position that the alleged dichotomy between health and the economy was a false one (FT, 11-11-2020).

Well before the publication of the letter, the task force had made efforts to convince the policymakers to tighten restrictions in view of soaring numbers, but also emerging empirical evidence that earlier measures could limit the scale, duration and severity of the wave (both in terms of health and economic costs, see for example Arnold et al (2022)). Nonetheless, and in contrast to the former two appeals, the November 2020 open letter was not signed by any member of the ncs-tf. In the public debate, some commentators voiced the concern that ncs-tf economists were silenced or at least restricted by the mandate’s communication strategy. While the issue of communication did play a role, the more important reason for the abstention was that the taskforce economists were convinced to be in a better position when addressing policymakers directly in an emotionally charged atmosphere.

Economic insights also found their way into teaching (see Brunetti (2021) for an example for Switzerland), executive education, public lectures and social media. Many economists in and outside the ncs-tf contributed to the public good by posting their slides or presentations online for easy access, or by engaging in discussions on social media.

## The right tools

In the last decades, few crises have had such a strong impact on virtually all economic decision-making as the COVID-19 pandemic. It became clear very quickly that the pandemic impacted both the supply and demand sides of the economy. Firms were forced to reduce their production and consumers' ability to consume dropped. The shutdown due to government-imposed mobility restrictions and personal decisions from individuals triggered the sharpest and deepest recession in the post-war period. In all countries, claims for unemployment insurance or short-term work soared to hitherto unknown levels.

The breadth of economic questions raised is well mirrored in the huge research output. What I am covering in this section is a personal summary of the economic tools that were in highest demand by policymakers and the public to shed light on the issues and help find policy options to lessen the impact of the crisis—or to speed up recovery.

With few exceptions, the economic expertise asked for by decision-makers or the public did not require complicated reasoning or modelling: Descriptive evidence, putting into perspective, explaining incentives and externalities, and in some cases ruling out nonsensical ideas. Economic principles proved to be very powerful in conversation with other sciences. The move of economic research towards more empirical questions in the last decades, and the provision of new data turned out to be very helpful during the pandemic.

### The power of (new) data

“The partial shutdown of the economy following the outbreak of the COVID-19 pandemic has highlighted the lack of measurements of economic activity that are available with a short lag and at high frequency” (Lengwiler, 2020, p. 1). What Yvan Lengwiler writes in this Journal was experienced by both policymakers and researchers. The usual measuring rod for macroeconomic performance, the GDP, was of little use in a rapidly evolving crisis. Indeed, most macroeconomic data is only published with substantial lags.

Economics was not the only field to suffer from a lack of data at the onset of the pandemic. Even more true for epidemiology and medical sciences, reliable and quickly available data is essential for evidence-based policy recommendations (that can subsequently be taken up or discarded by the political decision-makers). In fact, economics was in a better position than epidemiology, as not all advice is dependent on short-run data.

The shortage of data rapidly triggered efforts to close the gap. Economists seemed to thrive in this situation. Tellingly, what became the most important and reliable data platform during the crisis on a global scale, used by all disciplines and media outlets, *Our World in Data*, was founded and is still largely run by economists. Be it for epidemiological data, information on health measures or restrictions, the standardized way to record and display the data, as well as open access and easy use, facilitated international comparisons and analysis for single countries.

Turning back to macroeconomic indicators, fortunately, a number of economic time series data such as financial data, mobility indicators, and energy use are available relatively quickly and can be combined to mimic GDP. To cover all the efforts to generate such data would go beyond the scope of the paper, but a number of examples for Switzerland shall illustrate the successful quest for ways to measure the economic impact of the crisis.

As early as March 2020, ETH’s KOF published a High-Frequency Economic Monitoring Dashboard. In their paper, Eckert and Mikosch (2020) describe their daily compound indicators on physical mobility, sales activity, economic activity inside Switzerland, and international travel activity of Swiss residents. The encompassing activity indicator constructed from these data was subsequently made available for the interested public for download and visual inspection.

Even closer to an early indicator for the Swiss GDP, the “fever curve” developed by Burri and Kaufmann (2020), uses publicly available daily financial market and news data. The authors show that the measured fever is highly correlated with macroeconomic data and survey indicators of Swiss economic activity.

The SECO itself came up with a useful alternative to GDP within a very short period time. Their index of weekly economic activity (WEA, see Seco (2021)) provides rapid information about the growth of the Swiss economy combining information on nine indicators (air pollution, transaction, withdrawals, exports, import, electricity consumption, sight deposits, registered unemployed, net tonne-km). While the WEA cannot replace GDP it shows a high correlation with the growth of real GDP in Switzerland and supplements the existing data.

**Fig. 1:**
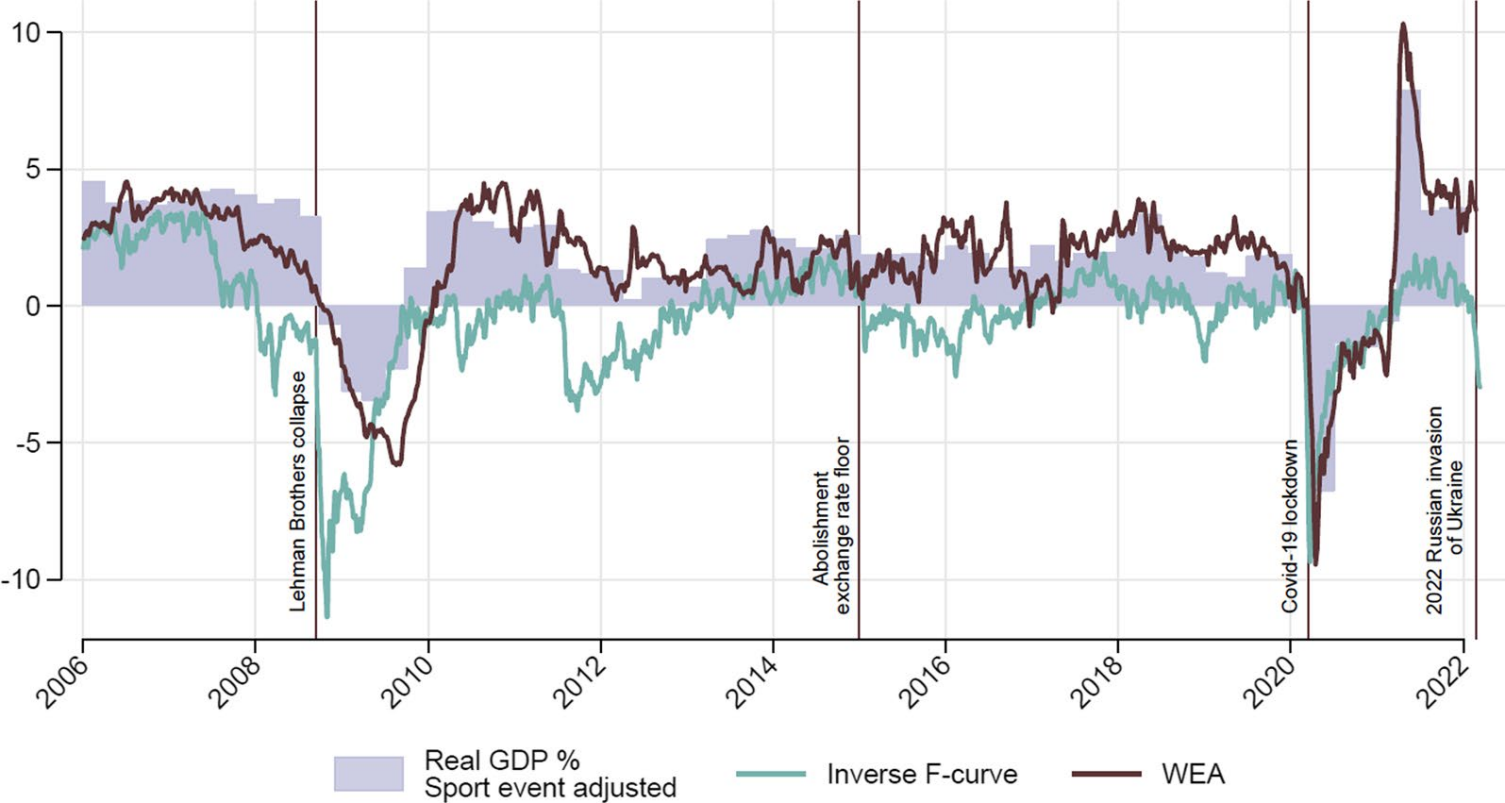
Measurement and alternative indicators of Swiss GDP, data sources mentioned in the main text

Figure 1 provides an illustration of how well the new indicators can mirror economic activity since 2006. SECO’s WEA (in brown) is able to track the GDP (bar graph, purple) very well and seems to offer even more granular insight on GDP movements. Burri and Kaufmann’s fever curve (inverse F-curve, light blue), based on financial data only, does a decent job in describing changes to real GDP.

On a more granular level, new data was made available to follow the course of specific sectors during the pandemic. Kraenzlin et al. (2020), for example, demonstrate regional shifts in Swiss retail payments caused by COVID-19. In applications, notably in the media, another project received quite some traction: Monitoring Consumption Switzerland (Brown et al. (2022), a joint initiative of the University of St. Gallen, the University of Lausanne–E4S, and private partners. The project uses aggregated and anonymized payment data to shed light on consumer spending in Switzerland and how this is impacted by the COVID-19 crisis. Its platform also offers various dashboards, fact sheets, further analyses, and comprehensive media reviews in four languages.

Apart from now-casting the pandemic, assessing the impact of the pandemic triggered interesting research with new data. Here are four examples of work that uses novel data to shed light on a range of questions.

Has the crisis sped up firm bankruptcies, or at the opposite end, have compensation measures proved to be too protective, preventing healthy creative destruction? An early study (Brülhart et al., 2020) estimates the effect of COVID-19 financial public support measures using a survey of self-employed workers and small business owners in Switzerland. They find that “objective” measures of lockdown affectedness and economic structure explain fairly well how businesses profited from support measures to cover labour costs.

Eckert and Mikosch (2022) explore the incidence of firm bankruptcies and start-ups in Switzerland based on unique register data and an idea borrowed from epidemiology: The authors apply the concept of excess mortality to assess the frequency of bankruptcies over time. In contrast to previous economic downturns, bankruptcy rates were substantially lower as compared to the pre-crisis period across most industries and regions. In the winter of 2021, bankruptcies rebounded strongly. Since the summer of 2020, the number of new firm formations has been significantly higher compared to the time before the crisis. This is also in contrast to the previous crises. The strong start-up activity is driven by industries where the pandemic induced structural adjustments.

While it was undisputed that the young generation was heavily restricted by confining measures, Goller and Wolter (2021) demonstrate that the recession in Switzerland triggered by COVID-19 ultimately remained without consequences for the apprenticeship market. The authors use daily search queries on the national administrative platform for apprenticeship vacancies from February 2020 until April 2021 as a proxy for the supply of potential apprentices.

Another fear, often voiced at the start of the pandemic, was that confinement measures would lead to additional stress that could ultimately be as damaging as the virus. To address this concern, Brülhart et al. (2021) used data from helplines, which offer a real-time measure of revealed distress and mental health concerns. Call volumes started increasing after the onset of the pandemic and peaked a few weeks later. Issues linked directly to the pandemic such as fear of infection, loneliness and concerns about physical health seem to have replaced rather than exacerbated underlying anxieties. Relationship issues, economic problems, and violence were found to be less prevalent than before the pandemic. The initial idea, first published with Swiss data as a blog entry (Brülhart & Lalive, 2020), was later extended to include 19 countries and subsequent waves of the COVID-19 crisis.

### The beauty of simple concepts: economic principles and back-of-the-envelope calculations

Even before the pandemic, basic economic concepts, such as opportunity costs, trade-offs, and externalities, had been far more useful than economists themselves may have perceived. And they are remarkably unfamiliar to many educated minds outside economics, in parts mirroring the lack of economic education in secondary schools in Switzerland.

During the pandemic, economic principles have not only been very helpful in discussions with other scientists and decision-makers; they also found their way into the public debate. One of the most powerful tools of economics is to spell out the costs of an action or a policy in terms of the trade-offs implied. Trade-offs were addressed in the public sphere early on. Should one save a few hundred elderly for billions (bn) of CHF output lost? Do restrictive measures do more harm than good, because the calculations do not account for relationship issues and psychological health?

When it comes to trade-offs during the pandemic, there have been some relatively easy ones such as the costs and impact of contact tracing. But most decisions involving trade-offs are not-so-easy ones, because choices entail externalities and long-term effects or behavioural changes. Almost all of the ncs-tf’s policy briefs discuss, and in some cases quantify, trade-offs. Table 1 lists the trade-offs discussed in a separate column. I will discuss the big and complex health–wealth trade-off in a separate section below.

Another basic insight in economics is that while markets are usually a good way to organize economic activity, the government can sometimes improve market outcomes. The ncs-tf itself would not have had a meaningful function if the authorities had not had ways to improve the situation with appropriate policies.

Among the many reasons for markets to fail, externalities were by far the most important one during the pandemic. Preventing infections through distancing or mask-wearing has private costs and social benefits, similar to contributions to a public good. If people only equated private benefits and costs, there would be insufficient distancing. Policies such as closings, cancellations, and restrictions on mobility addressed these externalities. The same applies to testing. If people only equated their private benefits and costs, there would be insufficient testing. As in many other countries, Switzerland followed the advice of experts to subsidize testing and later vaccines to overcome the implied negative externalities.

In addition to economic principles, simple back-of-the-envelope calculations proved extremely useful for public policy during the crisis. This should by no means diminish the desirability of precise indicators, empirical estimates, and careful modelling. But often simple comparisons were very effective in conveying the main message to policymakers and the public. Rough estimates can capture the magnitude of an effect, assess the plausibility of a finding, and rule out some potential explanations. Moreover, they are easy to explain and reproduce.

An early policy brief of the ncs-tf (Table 1, 10/05/2020), for example, pointed out that the costs of testing, tracing, isolation and quarantine (TTIQ) were much smaller than the costs of sick days, which in turn are much lower than the costs of closures. As a further example, let me add the simple back of the envelope calculation to quantify the loss in delaying the vaccination campaign. Starting from a yearly GDP of 720 bn CHF, the daily output is around 2 bn/day. A rough estimate of remaining restricting measures in January 2021 by the KOF was approximately 2%, or 40 million a day. Hence, the benefit of accelerating the vaccination campaign and shortening the closures by one day amounts to approximately 40 million CHF, which clearly exceeds any imaginable cost of the campaign. The ncs-tf policy brief (Table 1, 19/01/2021) offers a somewhat more detailed estimate and arrives at a lower bound of 25 million for a one-day delay in starting the vaccination campaign.

### Behavioural adjustments: voluntary or not so voluntary?

The impact of behavioural adjustments on decisions such as investment and consumption has long been understood in economics. Retrospectively, one of the more important contributions of economists in the public debate and in discussions with the policymakers was to point out that individuals react to changing circumstances even in the absence of mandates and restrictions.

However, it is complex to distinguish voluntary from involuntary restrictions and thus to disentangle the effects of the virus and the policies aiming at containing it. Whether people would have changed their behaviour without non-pharmaceutical interventions or not depends on the nature of the specific measure. Hard measures, such as lockdowns, would probably not have happened voluntarily. Softer measures, such as wearing a mask or social distancing could more easily happen on a voluntary basis. Last but not least, the relative importance of voluntary and involuntary adjustments is likely to change over time, making it harder to forecast the impact of measures.

In almost all countries, individuals restricted their mobility well before formal restrictions were in place. International research shows that behavioural adjustments were, to a large degree, responsible for the economic downturn in the first wave. Ignoring the negative impact of the international economy, authors estimate the share of the downturn due to voluntary measures in a range from 50 to 90% (Andersen et al., 2020; Aum et al., 2021; Goolsbee & Syverson, 2021).

**Fig. 2:**
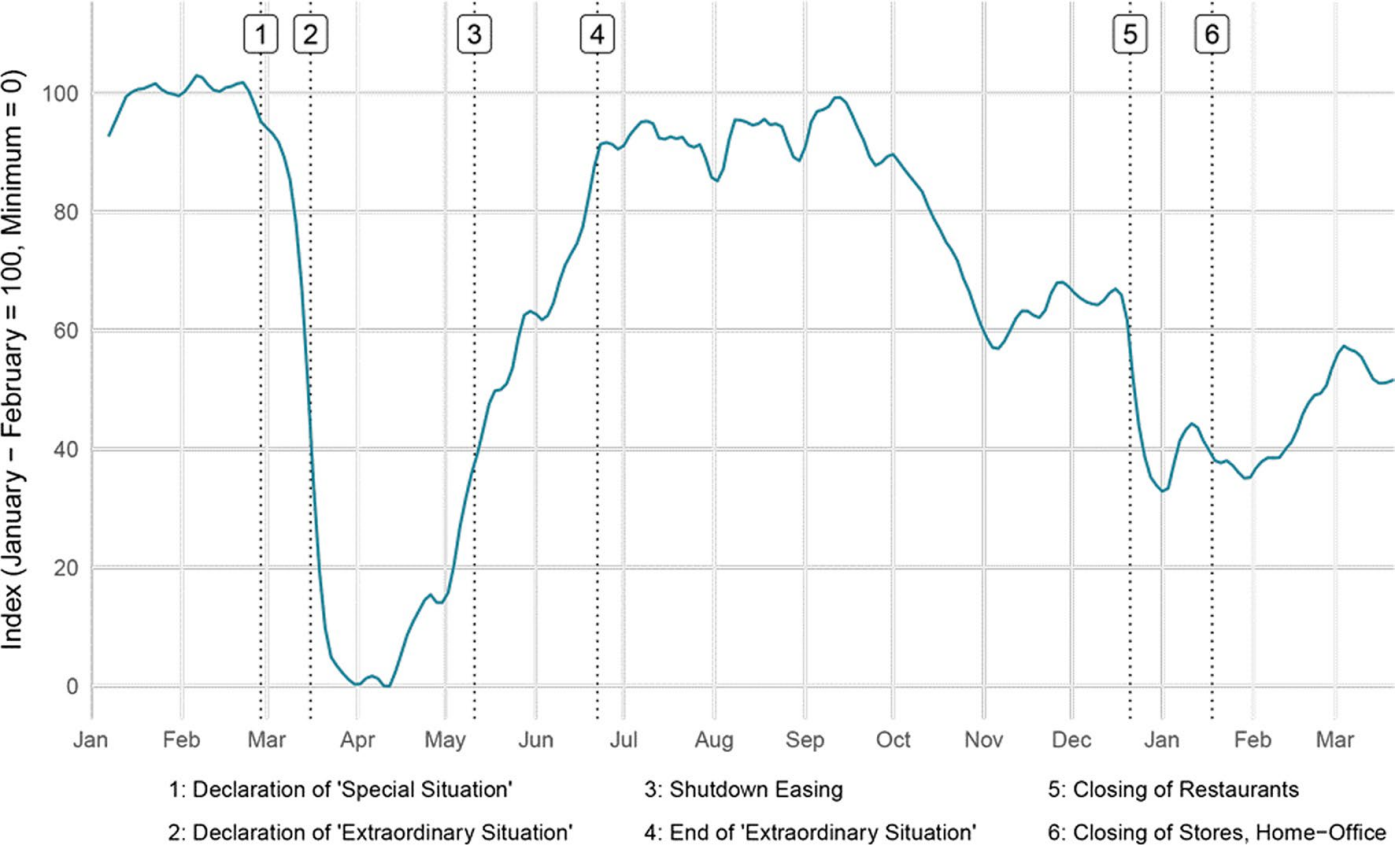
Mobility of the Swiss population during the pandemic, January 2020–March 2021 (KOF, 2020)

Figure 2 displays KOF’s mobility indicator during the pandemic’s first two waves. By the time the national shutdown in Switzerland was declared on 16 March 2020, KOF’s mobility index had already fallen to 40% of its pre-pandemic level. A decline in activity can be detected during the second wave, albeit in a much more reduced form, despite the fact that the second wave was more deadly and had a far higher virus circulation. The illustration also shows that experiences from one phase of a crisis can only be applied to later phases with caution.

### The big trade-off: health versus wealth

For policymaking, it is important not only to understand specific trade-offs, but also the multitude of costs generated by the pandemic—in both the medical and the economic domain. At least in the short run, government interventions create a trade-off between saving lives and preserving economic activity (or livelihoods), the so-called health–wealth trade-off.

Comparing economic and health costs and their benefits at an individual level is not uncommon, for example, when deciding to allocate scarce drugs or donor organs to patients in need or determining pay-outs of damages for death and injuries in legal claims. Usually, such comparisons include not only simple survival probabilities but also the number of life years at stake and their quality. The most common measure is QALY—quality-adjusted life years. One QALY equates to one year in perfect health.

At a macroeconomic level, however, interdependencies complicate the assessment to balance the benefits in terms of QALYs and costs of policies to mitigate the virus for a number of reasons. The first problem is that the correct counterfactual against which costs and benefits could be assessed is unknown. To estimate the true costs of restricting measures, we would need to compare the status quo with a situation without restrictions, but with the virus circulating freely.

Second, causal chains are often unclear. Economic costs not only arise because of restrictions, but also because of voluntary behavioural adjustments, as outlined above. The government may have mandated a stay-at-home order, but individuals might have stayed at home even in the absence of such a policy, be it for fear of the virus or for other reasons. Third, in a globalized world, there are large spillovers to all, even “non-affected” countries. The pandemic affects countries even in the absence of infections. The higher the incidence of the virus, the higher the likelihood of propagation of negative economic shocks, as export demand contracts and financial markets become more volatile.

Fourth, time lags and uncertainty about the evolution of the pandemic make it difficult to assess the trade-off. Moreover, trade-offs also depend on the assumptions about the availability of vaccines or better treatment options. All estimates are burdened with large degrees of uncertainty—probably much larger than during the financial crisis. Last but not least, economic costs also depend on the nature and effectiveness of compensation mechanisms taken by the government to alleviate the pandemic’s impact. Trade-offs, therefore, look very different in countries with fewer means or less efficient institutions to mitigate the economic damage of restricting measures.

In the early days, economists have come to call the trade-off between “health” and “wealth” the “double flattening problem” (Gourinchas, 2020). Flattening the virus spread curve with hard measures (such as lockdowns) depresses economic activity. However, economic policy can help to limit the economic damage (flatten the economic cost curve) to some degree and thereby ameliorate the health–wealth trade-off. This conceptual framework helped to understand the dynamics of the crisis but was ultimately too rigid to serve as a base for policy work.

Given the complexity of the trade-offs, it is not surprising that individuals and political decision-makers have struggled to understand the pandemic. Nonetheless, economic research has tried to shed some light on the trade-offs associated with the pandemic (see, for example, literature cited in Bütler et al. (2020)). The knowledge transfer from research into policy is more difficult. A humble goal is to educate decision-makers and the public about the limits to assess *ex ante* the impact of both health and economic measures. A second way is to come up with potentially simplifying trade-offs for particular situations in which political actions are assessed.

Such an assessment was explicitly requested by the Federal Council in the midst of the second wave. At its meeting on 18 December 2020, it asked the ncs-tf to present an economic analysis of the necessity and consequences of the measures decided so far by 13 January 2021. In their policy brief (Table 1, 19/01/2021), published a few days later, ncs-tf’s economists came to the conclusion that the far-reaching health policy measures in place in January 2021 were appropriate from a macroeconomic perspective. The analysis was based on heavy utilization of hospital capacities, significant excess mortality and the prospect that vulnerable people and later the entire population could be vaccinated relatively quickly. In such a situation, the duration of extensive health policy measures is limited, improving their cost–benefit ratio. The expert group also reiterated the need for adequately compensating lost income to minimize the costs of health measures for the private sector and underlined the benefits of accelerating vaccination campaigns.

The fact that the trade-off was numerically calibrated was quite remarkable and courageous, given the large degree of uncertainty around the assumptions underlying the scenarios. A few weeks later, it turned out that the realized outcome of the pandemic’s course was better than anticipated. The discrepancy between the scenarios and the actual outcome gave rise to a controversy over the role of the ncs-tf in decision-making both in the public and in politics. Once more, it proved to be difficult to convey the message that risks have to be assessed *ex ante*, and not *ex post*.

What can safely be stated is that the COVID-19 pandemic is a common public health and economic shock. The exact nature of the trade-off between the health and wealth in a pandemic depends strongly on policy spillovers and behavioural responses of firms and individuals. As a consequence, the coordination of economic policies and public health measures is key. (Which also implies that the composition of the ncs-tf proved to be meaningful along these considerations).

### Economic policy advice, more traditional

Economists in the ncs-tf were primarily engaged in providing analysis and advice to its principals, such as the Federal Office of Public Health, and peers within the ncs-tf, especially during the first wave. But they also ventured out into more traditional types of policy recommendations, together with other academic economists.

Around the world, economists pointed out the importance of targeted support measures from an early stage in the pandemic. If individuals lose their job as a consequence of a lockdown, for example, their effective well-being crucially depends on income replacement programmes and other measures taken by the government. Support measures can also be viewed as a way to reinforce sanitary measures to fight the virus. If individuals and firms are insured from income losses due to closures and restrictions at least partially, there is less need to engage in banned activities that potentially boost infections.

However, public support measures can be more or less effective: A straightforward comparison of the cost of support measures and GDP losses between different countries shows striking differences (Schaltegger & Mair, 2021). For example, while Austria and Switzerland had similar health outcomes in terms of mortality rates and both countries spent similar amounts of government aid per capita, Austria’s loss in GDP was more than twofold the one of Switzerland’s. The USA, on the other hand, spent 2.5 times as much as Switzerland on support measures for a similar fall in GDP. What caused these differences is difficult to pin down, but it illustrates that the relevant question is not only *how much* to spend, but rather *what* to spend these scarce resources on.

In Switzerland, there was little discrepancy between the economic measures taken by the government and the recommendations of academic economists during the first phase of the pandemic. The speedy measures were unprecedented in magnitude, administrative simplicity, and outstanding in international comparison. Short-term work was expanded rapidly, and credit lines were made available in an unbureaucratic manner to affected businesses within a few days, thanks to an unprecedented collaboration between the SECO, the Swiss National Bank, and commercial banks. I am convinced that the speedy reaction of the government boosted confidence and helped businesses find the energy to deal with the real challenges and not bother with the financial situation only.

While underemployment of labour was adequately taken care of by the unemployment insurance, underemployment of capital was trickier to compensate for in the absence of an established insurance mechanism. A number of proposals by economists dealt with the issue. An early ncs-tf policy brief (Table 1, 01/05/2020) tackled the problem of commercial rents for businesses affected by closures. The economists suggested that the government should match rent abatements to incentivize landlords not to dissolve rental contracts and help both parties to reach a mutually accepted solution. Another policy brief (Table 1, 10/11/2020) dealt with the fiscal support to capital owners in the second wave. It suggested to reactivate the successful COVID-19 credits of the first wave, with the option to convert the loan into a fond perdu support if needed.

As the pandemic progressed and government involvement stayed high, the question of how to repay the government debt resulting from the COVID-19 crisis became more important. The huge amount of money spent on compensation measures, but also warnings issued by Switzerland’s finance minister and business representatives, sparked a lively debate both in the public and among economists. The challenge is to reduce newly accumulated debt successfully without choking off the prospects for a speedy recovery. In a policy brief from May 2020 (Table 1, 20/05/2020), ncs-tf economists discussed the different options, among others their preferred one of a debt repayment over a longer time period than 6 years (i.e. 30 years).

There were also proposals for the second part of the challenge, boosting economic growth. The policy trade-off faced during this period was to adequately support affected businesses without keeping alive non-viable projects (aka zombies). One suggestion by ncs-tf economists (Table 1, 03/08/2020) was to tackle weak investment with an adjustment to the COVID-19 credit programme. In a new or prolonged programme, loans could also be used for investment, not just operational costs.

## Conclusions: learnings for future public policy work

*“I am proud of being an economist. As a profession, we have responded to the challenge with ingenuity and imagination, drawing on our vast array of tools. I suspect that, collectively, we never created so much new knowledge in so short a time.” (*Wyplosz, 2021*, p. 2)*

I agree with Charles Wyplosz’ remark in the latest volume of *Covid Economics*—with some qualifications. Economic tools proved useful and new data sources were quickly made available for public use. Economists directly engaged in task forces helping to fight the pandemic—often without reimbursement, they participated in the public debate and interacted with decision-makers in ways hardly seen before. The question is whether the degree of involvement in important societal questions can be maintained in the future.

Before the crisis, complaints abounded that economists did not produce applicable research or—in case they did—were not able to translate their own research into comprehensible knowledge for society. The COVID-19 pandemic has shown that this is not the case. As in other fields, many academic institutions and their academic economists contributed to a better understanding of the pandemic’s economic impact and provided input for decision-making.

The complaint that academic research in normal times is bypassing social demand is not entirely unfounded, though. Important subjects are not dealt with because there are no laurels to be had. For example, when an allegedly similar question has already been answered (albeit for a different country), or when there is no interesting identification strategy to tease out the causal effect of a policy.

I want to add two additional observations that I found remarkable during the last two years. First, there was quite some discrepancy in tone in the public debate—be it in traditional or social media—between economists who were officially involved in task forces (ncs-tf and other working groups) and those without such affiliations. The latter appeared less apologetic and more critical of political decision-makers, interest groups and media outlets. This was the case for both economists who advocated for harder measures and those who objected to more stringent measures and restrictions. It was feared that ncs-tf members were silenced or at least restricted by the mandate’s communication strategy. A more benevolent interpretation is that working together with a broad range of other fields and talking to decision-makers both in the public and private sphere facilitates mutual understanding and leads to a more nuanced assessment of controversial questions.

The second observation concerns the interdisciplinary aspects of economic research, be it applied or theoretical. Despite the encompassing nature of the crisis, there has been relatively little interdisciplinary research. Even among the policy papers, single-disciplinary work dominates. Even less interdisciplinary work can be found when looking at research papers. This is not surprising given the current incentives to publish. However, the lack of interdisciplinary papers does not necessarily mean that the interdisciplinary dialogue did not take place. On the contrary: the crisis seems to have increased the dialogue between different fields. What strikes me as more critical are economists venturing out into other disciplines—epidemiology, for example—without an adequate placement into context by the respective discipline. I sometimes wished academic economists were a bit more self-critical and humbler in their assessments.

Some thoughts on letting the increased engagement stick and keep on producing knowledge for society. It is very unlikely that the ad hoc involvement during the crisis, in which many scholars lacked the necessary support from their university, is an optimal model. As with other scientists, economists do produce applicable research but may find it difficult to get credited for the additional effort. In the absence of spillovers to publishable research, applied work jeopardizes promotions and the chance to participate in teaching reductions or other benefits.

Another issue is that personalization and scandalization of the media discourage academics from making their research results accessible to a wider audience. An even greater hurdle is that spending time and energy on science communication is too often frowned upon by fellow scientists. In 25 years of participating in recruiting committees, I have rarely seen colleagues speak up on behalf of an applicant who had traded off a fraction of his research output for educating the public.

What seems like the obvious solution, the division of labour—some conduct research aimed to be published in reputable journals, others carry out applied research and speak in public -, leads astray for a number of reasons. Even "common sense" in economics must ultimately be based on a well-founded understanding of causal relationships. Own peer-evaluated research remains an important anchor also for public intellectuals. Academics who are well-rooted in the international research community represent (economic) policy advice with much more authority and credibility. Often, theorists and basic researchers are moving to more applied fields only later in their career. In addition, universities and research institutions offer a continuous exchange with other researchers and challenging students.

Universities and those looking for economic knowledge would be well advised to find models that allow scholars with a genuine interest in policy work to take trips out of the ivory tower. One option is to credit academics for public engagement under well-specified conditions. Another one would be extended leaves of absence with a return guarantee in case economists serve in public offices or engage in other types of knowledge transfer. This practice, long established in the USA, guarantees that application-oriented researchers can rely on the freedom of research, which ultimately forms the basis for good ideas to thrive.

If I had to summarize the role of economics and academic economists in Switzerland, I would stress the importance of economic ideas as well as the desirability of a continuous exchange of ideas between academia and decision-makers in both the public and private sphere. As economists, we were aware of the importance of good data on which decisions can be based, but we have probably underestimated the power of simple economic ideas and tools. That many Swiss economists were experienced both in collaborating with the administration and in communicating to the public proved to be a clear plus during the pandemic. We should find ways to help these advantages stick.

## Data Availability

No proprietary or original data, data sources for supporting plots are mentioned in the text.
